# Bite Wounds and Dominance Structures in Male and Female African Spiny Mice (*Acomys cahirinus*): Implications for Animal Welfare and the Generalizability of Experimental Results

**DOI:** 10.3390/ani14010064

**Published:** 2023-12-23

**Authors:** Justin A. Varholick, Gizelle Godinez, Ashley Jenkins, Sarim Mobin, Malcolm Maden

**Affiliations:** 1 Department of Biology, University of Florida, Gainesville, FL 32611, USA; 2 Department of Molecular Genetics and Microbiology, University of Florida, Gainesville, FL 32610, USA; 3 McKnight Brain Institute, College of Medicine, University of Florida, Gainesville, FL 32610, USA; 4 Department of Psychology, University of Florida, Gainesville, FL 32611, USA; 5 Genetics Institute, University of Florida, Gainesville, FL 32610, USA

**Keywords:** bite wounds, fight wounds, social dominance, aggression, animal welfare, sex differences

## Abstract

**Simple Summary:**

Animals often inflict bite wounds in laboratory housing, compromising animal welfare and, potentially, experimental results. For example, 1.5–14% of male laboratory mice (*Mus musculus*) obtain bite wounds over the course of a year. Recently, spiny mice (*Acomys cahirinus*) have become more popular in laboratory research for their regenerative healing and menstrual cycling, but we noticed frequent severe wounding in males and females in our colony—likely related to their relatively weak skin. Thus, we recorded the frequency and severity of bite wounding in our colony for one year and found that 46% of spiny mice had at least one bite wound, regardless of sex. The bite wounds in females were less severe, with single wounds on the rump, while males more often had multiple wounds on their anterior and rump. In a separate study, both sexes established stable dominance structures, with most of the fights occurring at the start of the active/dark cycle. Aged animals greater than 22 months old were never observed fighting, and the study on bite wounding suggested they also do not inflict bite wounds. These results have implications for both spiny mouse welfare and experimental results using spiny mice since repeated physical trauma can affect healing and menstrual cycling in mice and humans.

**Abstract:**

Bite wounds due to aggression in male laboratory mice (*Mus musculus*) are a major welfare concern, often leading to attrition, chronic activation of the innate immune system, and significant impacts on the experimental results derived from the use of these animals as models. Bite wounding within the home-cage of spiny mice (*Acomys cahirinus*)—a valuable research model for wound healing and menstruation—is poorly characterized. While we have anecdotally observed frequent bite wounding in *Acomys*, the frequency of aggression within the home-cage, the severity of the bite wounds, and the types of dominance structures remain unstudied. Here, we report that 46% of *Acomys* cages in our colony had at least one bite wound over the course of a year and that same-sex pairs fought in the home-cage 10% of the time during their dark/active phase. Both sexes inflicted wounds and frequently engaged in agonistic behaviors, even with stable dominance structures. We found that females inflicted less severe bite wounds in same-sex housing. Also, aged females in same-sex pairs were never observed fighting, and no bite wounds were observed in aged *Acomys*. These results suggest that we should consider whether bite wounding negatively impacts our experimental results since physical trauma is known to alter menstrual cycling and healing.

## 1. Introduction

**Figure 1:**
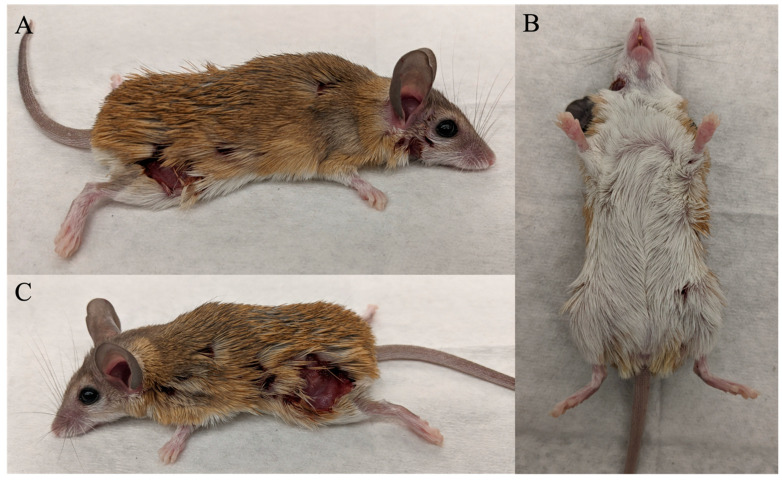
An *Acomys* with severe wounding. This animal was euthanized due to severe wounding. (**A**–**C**) are photographs of the same animal. (**A**) the right lateral side of the animal, with a large superficial wound on the rump, with a smaller wound to the right of that, another wound near the shoulder-blade, a wound on the nape under the ear, and a small wound under the eye. (**B**) The ventrum of the animal with the nape wound visible on the right-side of the animal and a small wound at the base of the left leg. (**C**) the left lateral side of the animal, with a small wound behind the ear, another small wound on the shoulder blade, and a large irregular wound on the left posterior flank/rump, deep with exposed muscle. No wounds were present on the tail.

African spiny mice (*Acomys* cahirinus) are a remarkable animal model in biomedical research, given their ability to heal injuries via scar-free tissue regeneration [1,2]. Additionally, *Acomys* have been used in studies of menstruation, social behavior, precocial development, and diabetes [1,3,4,5]. Unlike other laboratory rodents like mice (*Mus musculus*) and rats (*Rattus norvegicus*), standards for housing and care of *Acomys* are less well defined and are based on existing standards for mice and rats. Within these housing conditions, we observed abnormally severe wounds due to agonistic interactions (Figure 1). Agonistic behavior leading to wounding is associated with diminished animal welfare and compromised experimental results in *Mus* [6]. Severe wounding may be unavoidable in *Acomys* since their skin is 20 times weaker than *Mus* and therefore prone to tearing [1]. Although *Acomys* can effectively heal these severe wounds via tissue regeneration, bite wounding due to agonistic behaviors should be minimized to promote good welfare. Moreover, studies in *Mus* suggest that bite wounds can alter the innate immune system [7], which is central to promoting scar-free tissue regeneration [8]. Physical trauma can also lead to irregular menstrual cycles (e.g., polymenorrhea or amenorrhea) [9]. Given the link between bite wounding due to aggression and the potential modulation of the innate immune system and menstrual cycling, it is likely that experimental results derived from their use within these contexts may be idiosyncratic.

While all captive group-housed animals are known to fight, the incidence of bite wounds or injurious wounds from fighting is poorly characterized for laboratory animals. Most studies are limited to *Mus*, where 1.5–14% of animals are wounded by their cage-mates [10,11,12], the majority of which are male [6,13]. Research on male mice has demonstrated that altering the home-cage environment by adding or removing structural features may promote stable dominance structures, thereby reducing aggression [14,15]. The social dominance structures within the cage are often linked with the frequency of bite wounding and aggression. Stable dominance structures where animals maintain their ranking are common in same-sex groups of lab *Mus* [16,17]. However, some groups of *Mus* within the laboratory setting display unstable dominance relationships where individuals can unpredictably switch ranks during sudden fights [18]. When bite wounding does occur, wounding is often located at the rump or tail of all cage-mates except the aggressor [19]. Severe wounds are often discussed with a veterinarian, and the animal may be euthanized as a humane endpoint [20]. Thus, the frequency of fighting, sex differences, and dominance stability are important factors when considering bite wounding in laboratory rodents.

Studies on *Acomys* suggest that both males and females frequently fight, but no studies to date have measured bite wounds or dominance stability [21,22,23]. Early studies on *Acomys* note that they are precocial, with small litter sizes, and that mothers, fathers, and other animals in the group help rear the young [24]. They are also known to live in social groups for up to 3 to 4 years in captivity [25]. Specific studies on their agonistic behavior determined that both sexes are involved in agonistic encounters, contrasting with sex-specific male fighting observed in *Mus* [21]. These studies note that biting and counterattacks are rare in *Acomys*, and their agonistic behaviors mostly include chasing, fleeing, and displacement. The agonistic repertoire also excludes freezing or subordinate posturing, which again contrasts with many other rodents [21,26,27,28,29]. Also, in mixed-sex housing, females direct most attacks toward males [21], suggesting *Acomys* are more matriarchal than *Mus*. This attack behavior by females toward males appeared unrelated to postpartum aggression, which is commonly reported in other rodent species.

We are not aware of any specific studies on the stability of their dominance relationships or the severity of bite wounds in *Acomys*. Therefore, the aim of the current study was to determine the incidence and severity of bite wounding in our colony of *Acomys* and determine the stability and structure of their dominance relationships. This aim was carried out across two separate studies. The incidence and severity of bite wounding were studied by inspecting animals in the home-cage each weekday except holidays for one year, following a protocol similar to previous studies [11]. Animals were only disturbed or removed from their home-cage if bite wounds were observed. During wound inspection, wounds were scored using a severity score sheet modeled after the Lund–Browder burn wound chart for humans [30] and the ulcerative dermatitis scoring system for mice [31,32] to provide objective scores for later comparisons. In a separate study, the stability and structure of dominance relationships were measured in established adult groups over three consecutive weeks. The general incidence of agonistic behavior per group was measured for three consecutive nights each week using one-zero sampling. The dominance ranks, stability, and structures were determined following two 10 min observations at the start of each night for three consecutive nights for each week, using focal animal sampling.

## 2. Materials and Methods

### 2.1. Ethics Declaration

The experimental protocol was approved by the University of Florida Institutional Animal Care and Use Committee (protocol #201807707 and #202110500). All methods of this observational study were performed in accordance with the guidelines of the Guide for the Care and Use of Laboratory Animals [33].

### 2.2. General Housing

All *Acomys* were kept in a 14:10 light/dark cycle with lights on at 06:00. Temperature was maintained at 27 ± 3 °C and humidity around 50%. Animals were housed in either Techniplast GR1800 double-decker cages (floor area: 1862 cm^2^) or NexGen Rat 1800 cages with the addition of a second level (floor area: 1800 cm^2^). This difference in housing was due to cage maintenance issues during the experiment. All cages contained 2 cm deep of aspen wood chip bedding, and animals had ad libitum access to standard chow (Teklad 2918) and tap water. A special diet was provided once per week as determined by the veterinarian (e.g., grapes, mealworms, carrots, broccoli, and sweet potatoes). *Acomys* were provided with two shelters (Bio-Serv, Flemington, NJ, USA, red Rat Retreats) and multiple manipulada (i.e., a nylabone, a wood gnawing block, and a chewing stick).

### 2.3. Study 1

#### Incidence and Severity of Bite Wounding

**Figure 2:**
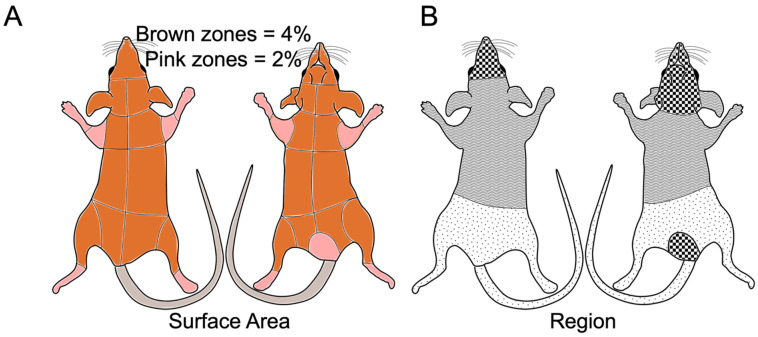
Wound severity reference. (**A**) Reference diagram for estimating the percent surface area of the bite wound injury. Each zone is outlined in black. Each brown zone is ~4% of the surface area, while each pink zone is around 2% of the surface area. (**B**) Reference diagram for determining the region of the bite = wound injury. The dotted region represents the posterior body, the wavy region represents the anterior body, and the checkered regions represent the face and genitals, which are particularly sensitive to injuries as determined by burn wound injuries in humans [30].

For one year, all animals in our colony were observed every weekday, excluding holidays, for the presence of bite wounds. Observations were made from outside the cage, and animals were only handled if bite wounds were present. A total of 122 animals were monitored across 47 cages, which included 10 cages of mixed-sex breeders (ages 35 days to 669 days), 22 cages of same-sex males (ages 24 days to 184 days), and 14 cages of same-sex females (ages 23 days to 382 days). Mixed-sex breeders were in groups of 2 to 5, while same-sex cages were in groups of 2 to 4. The number of animals in a cage was determined by the availability of animals on the day of weaning, which was highly variable since *Acomys* have small litter sizes of 1 to 4 pups (normally 2) after a 40-day gestation [5]. All animals were housed together since weaning. Animals were no longer observed once they entered an active experiment. Singly housed animals were excluded from this study. On average, animals were observed for a total of 135 days. When an animal with a bite wound was observed, we derived a scoring system following [30,31,32]. Four metrics were measured on a 0 to 3 scale, with 0 being no lesion and 3 being the most severe wound value for a total score of 12 (Table 1 and Figure 2). The surface area percentages in Figure 2 were determined from a single 4-month-old *Acomys* that was scanned using a micro-computed tomography (micro-CT) scanner, which was then segmented to determine the relative % surface area of each annotated area. A veterinarian determined a humane endpoint from bite wounds independent of the severity score, but post hoc comparisons found that a score of 10 or higher was marked as a humane endpoint. In most cases, veterinarians determined that scores of 8 or lower were monitored for healing or successive wounding. Scores of 9 were separated to avoid a humane endpoint. Successive wounding across multiple weeks/months also led to separation.

### 2.4. Study 2

#### 2.4.1. Subjects

In the second study, we observed the behavior of 9 groups housed in 4 different pair housing groups since weaning: 3 cages of young female pairs, 3 cages of young male pairs, and 3 cages of aged female pairs. Unfortunately, aged male pairs were not available in our colony. The young animals were 90 to 270 days, while the aged animals were 700 to 1034 days of age.

#### 2.4.2. General Incidence of Fighting Behaviors

Video recordings for 10 h were taken for 3 consecutive days per week for 3 weeks, creating 9 timepoints of data, or 90 h per cage. One-zero sampling of agonistic and huddling behaviors (c.f., Table 2) was performed during the first 15 min of every hour using BORIS behavioral coding software (v7.12.2) [34]. If the 15 min overlapped with the focal animal sampling, the next 15 min period during the hour was chosen. A subset of young animals (2 groups of young female pairs, 1 group of young male triads, and 1 group of young male pairs) was used to measure the representative frequency of activity, agonistic behavior, and huddling behavior across 24 h for all 9 timepoints. This determined that *Acomys* were most active at night (Figure S1). All animals were used to determine the frequency of activity, agonistic behavior, and huddling behavior nightly (dark cycle, 20:00 h to 06:00 h) across the 9 timepoints. All recordings were taken one day following cage changes or special diets since they can affect fighting in the home-cage (see the experimental timeline in Table S1 for more details). Inter-rater reliability of the behavioral recordings was good, with a 0.96 kappa for 10% of the data, and an intra-rater reliability of 0.92 for 5% of the data. Note that due to the COVID-19 pandemic, no video recordings were taken for old female groups during the third week.

#### 2.4.3. Dominance Ranks, Structures, and Stability

Here, focal animal sampling was used to determine dominance ranks, structures, and stability. All focal animal sampling recordings were taken on the same days as the one-zero sampling recordings, again totaling 9 timepoints across 3 consecutive weeks. Two 10 min recordings were taken at the start of each night (20:00 h to 23:00 h), totaling 180 min per cage. The total frequency and duration of offensive and defensive agonistic behaviors (Table 2) were recorded for each individual animal using BORIS behavioral coding software (v7.12.2) [34]. Individuals were identified by fur shavings to the hind limbs or forelimbs. The mean kappa for inter-rater reliability was 0.87 for 10% of the data, and for intra-rater reliability was 0.96 for 5% of the data.

Social dominance status, dominance structure, and stability were determined by the agonistic behaviors in the focal animal sampling. The data were either grouped together across all 9 timepoints to determine the cumulative dominance or for each week to determine the weekly dominance. For either cumulative dominance or weekly dominance, the social dominance status was determined by a cumulative David’s score calculated using the ‘compete’ package v0.1 [35] in R (v4.3.1). The cumulative David’s score is an index of the proportion of wins adjusted for the strengths of their opponents (i.e., how often their opponents win or lose) [36]. The social dominance status was determined by ranking David’s scores from highest (i.e., the dominant) to lowest (i.e., the subordinate) within the cage groups. Animals that did not engage in agonistic behavior had an unmeasurable social dominance status.

To determine the dominance structure, a directional consistency (DC) index was used for pairs of *Acomys*. The DC index was calculated using the ‘compete’ package v0.1 [35] in R (v4.3.1). A score of 1 indicates the relationship is asymmetric with all offensive agonistic behaviors from one animal (i.e., the dominant animal), while a score of 0 indicates the relationship is symmetrical with equal numbers of offensive behaviors between the pair [37].

Stability was assessed by determining the weekly social dominance status via the weekly David’s scores and noting the frequency of weekly agonistic interactions between groupmates. Groups were categorized as either “static with frequent weekly interactions” (Static Frequent), “static with infrequent weekly interactions” (Static Infrequent), “dynamic with frequent weekly interactions” (Dynamic Frequent), or “dynamic with infrequent weekly interactions” (Dynamic Infrequent). Static referred to the dominance status from David’s score not changing across the three weeks, while dynamic referred to the dominance status from David’s score changing across the three weeks. Frequent referred to the groupmates engaging in agonistic behaviors every week of observation, while infrequent referred to groups where agonistic behaviors were not observed for one week or more (i.e., David’s score of zero). Notably, some groups never engaged in agonistic interactions and were therefore labeled as “Unmeasurable”. Due to the COVID-19 pandemic, no video recordings for the third week were collected for the aged females, who were never observed to engage in agonistic interactions during the first two weeks (see Results).

### 2.5. Statistical Analyses

Two sets of statistical tests were run, one set for each study. All tests were completed in R (v4.3.1) [38]. For the bite wounding study, the data were not normally distributed, thus requiring non-parametric tests. A Kruskal–Wallis test was used to compare the “total wound score” across housing conditions (i.e., males, females, and breeders) and when comparing separate wounding metric scores (i.e., area, character, deepness, and region) for each housing condition. A *p*-value ≤ 0.05 was set as the critical threshold for significance. Post hoc comparisons of significant effects were followed by Dunn’s test with *p*-values adjusted for multiple comparisons using the FSA package [39].

For the dominance study, a stepwise approach was taken to analyze the incidence of fighting behavior across 9 timepoints. First, a linear mixed-effects model using the lme4 package [40] was carried out to compare the housing groups across the 9 repeated timepoints, with separate models for each behavior. The data were not normally distributed; thus, a constant was added to the outcome measure and log-transformed. This mixed-effects model revealed that there was no significant effect of timepoints for any behaviors, suggesting a simpler model would be appropriate. Thus, we computed the mean values for each cage group across the 9 timepoints and used these values in a Kruskal–Wallis test. Again, a *p*-value ≤ 0.05 was set as the critical threshold for significance. Post hoc comparisons of significant effects were followed by Dunn’s test with *p*-values adjusted for multiple comparisons using the FSA package [39].

## 3. Results

### 3.1. General Incidence and Severity of Bite Wounding

**Figure 3:**
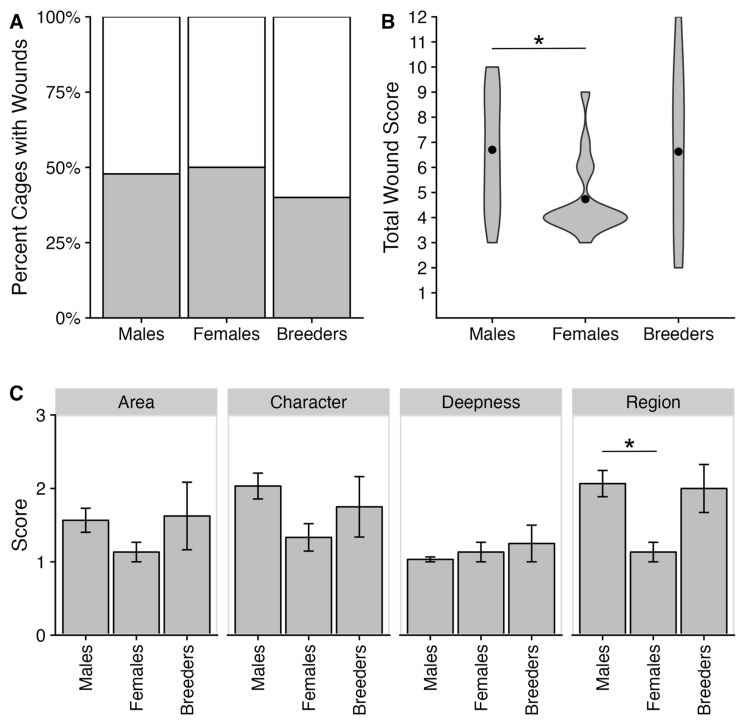
Bite wound incidence and severity. (**A**) Stacked bar graph of the percent of cages with one or more wounds across the year. The grey color represents wounded animals, while the white color represents no wounds. (**B**) Violin plot of the total wound score for each housing condition. Females had significantly lower total wound scores than males (Z = −2.67, padj = 0.022). (**C**) Panel graph of bar plots for each wound severity metric for each housing condition. Each bar refers to the mean, while error bars refer to the standard error of the mean. Females had significantly lower region scores than males (Z = −3.14, padj = 0.005). The * refers to a significant effect with a *p*-value ≤ 0.05.

After monitoring for bite wounds in our colony for one year every weekday, we determined that 46% of cages had an animal with a bite wound in the cage. This estimate was similar for mixed-sex housing (i.e., breeders), same-sex females, and same-sex males (Figure 3A). The mean total wound score was around 6 out of 12, where 0 represented no wounding, and 12 was the most severe wounding possible (Figure 3B). There were significant differences in wound severity between the groups (χ^2^(2) = 7.23, *p* = 0.027). Same-sex females had significantly lower scores than males (Z = −2.67, padj = 0.022) with a mean value of 4.73, indicating that the bite wounds inflicted in females were less severe than same-sex males. Comparing the separate wound metrics, there were only significant differences between the groups in the region of the bite wounds (χ^2^(2) = 10.47, *p* = 0.005) (Figure 3C and Table S2). Again, same-sex females had significantly lower region scores than same-sex males (Z = −3.14, padj = 0.005), indicating that females more often had one posterior wound while males more often had multiple wounds in the anterior and posterior. We also measured whether age was related to whether an animal was wounded and found no significant difference (χ^2^(1) = 0.031, *p* = 0.86), nor did we find a significant difference between age and severity score (R^2^ = 0.153, *p* = 0.282) (Figure S2).

### 3.2. General Incidence of Fighting Behaviors in Same-Sex Pair Housing

**Figure 4:**
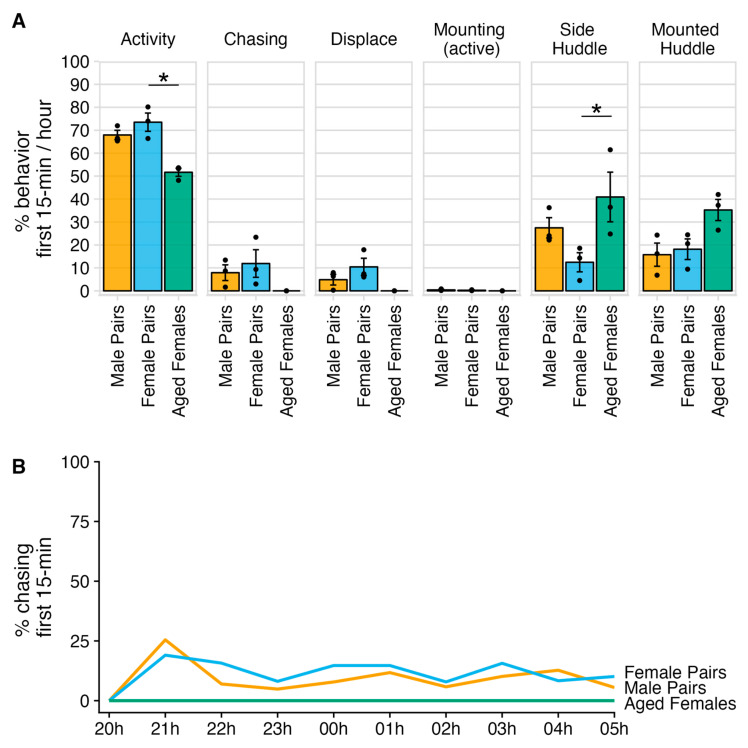
Agonistic and huddling behaviors observed during the night. (**A**) Panel of bar plots for each measured behavior for the different housing conditions. Bars represent the mean percent of the behavior observed in the first 15 min of every hour for each housing condition; error bars represent the standard error of the mean; and individual points represent the mean value per cage. Aged females had significantly lower activity scores than female pairs (Z = −2.385, padj = 0.05) and side-huddled significantly more than female pairs (Z = 2.534, padj = 0.034). (**B**) Time-series line graph of chasing for the different housing conditions across each hour of the night. Intersections between the *x*-axis breaks and line represent mean percent of chasing observed in the first 15 min for the respective hour. The * refers to a significant effect with a *p*-value ≤ 0.05.

In our second study, one zero-sampling for the first 15 min of each hour of the night was used to determine the general incidence of fighting and huddling behaviors in pair housing. The study was composed of three groups of same-sex male pairs, three groups of same-sex female pairs, and three groups of same-sex female pairs that were aged (i.e., 23 to 34 months). Aged male pairs were not present in our colony. Most cage groups were active for more than 50% of the night and only engaged in agonistic behaviors for around 10% of the night (i.e., chasing, displacement, or active mounting) (Figure 4A). We only found that activity and side-huddling was significantly different between housing groups (Activity: χ^2^(2) = 5.956, *p* = 0.05; Side-huddle χ^2^(2) = 6.489, *p* = 0.039) (Figure 4A and Table S3). Aged females were significantly less active than younger female pairs (Z = −2.385, padj = 0.05) and side-huddled significantly more than younger female pairs (Z = 2.534, padj = 0.034). Agonistic behaviors were most prevalent one hour after the lights turned off and generally cycled every 3 h (Figure 4B). Fighting in the aged female pairs was never observed.

### 3.3. Cumulative Dominance Structures in Same-Sex Pair Housing

In our second study on the incidence of fighting behaviors in pair-housing, focal animal sampling for two 10 min periods at the start of the dark cycle was used to determine the dominance structures of our groups. For this analysis, we measured the cumulative dominance structure across all three weeks of observation—summing the outcomes of all dominance interactions regardless of the week. All male pairs had a clear asymmetric structure where the dominant consistently chased or displaced the subordinate, who consistently yielded rather than counter-attacked (Table 3). Two-thirds of the female pairs had the same asymmetric structure, with one cage group having a more symmetric relationship with counter-chases by the subordinate. Aged females, however, were never observed fighting during these windows of observation; thus, we were unable to measure the structure of their dominance.

### 3.4. Stability of Weekly Dominance Structures in Same-Sex Pair Housing

**Figure 5:**
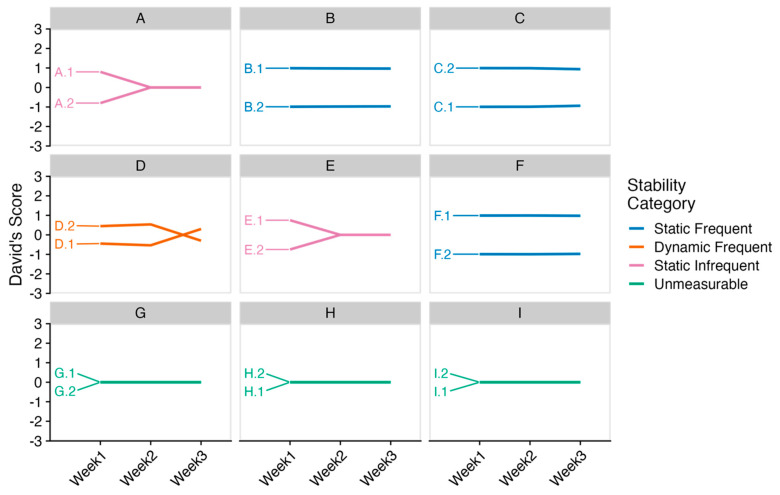
Stability of dominance behavior across three weeks. Panel of line graphs of David’s scores across three weeks for each cage, denoted by the letter. Each line represents an animal, which is labeled the cage letter (e.g., A) and animal number identification (e.g., A.1). The cage letters match those in Table 3. The top row is male pairs, the middle row is female pairs, and the bottom row is aged females.

Using the same data from the cumulative dominance structure analysis, we divided the observations into weekly dominance structures to measure the stability of the structures. All male pairs (i.e., cages A, B, and C) had a stable asymmetric structure, but one pair only fought during the first week of observations (Figure 5). Two-thirds of the female pairs (i.e., cages D, E, and F) had stable asymmetric structures, with one of them only fighting during the first week. The cage that had an unstable structure was the symmetric cage of females (i.e., cage D) from the cumulative dominance structure analysis, where the pair seemed to have switched dominance ranks in the third week of observation. Again, the aged female pairs (i.e., cages G, H, and I) were never observed fighting, and thus, we were unable to measure the stability of the dominance structure, albeit it did not change across the weeks. Raw values for the frequency of fighting for each cage for each week can be found in the Supplementary Materials (Table S4).

## 4. Discussion

The first study indicated that male and female *Acomys* fight and inflict bite wounds within the home-cage, and the second study suggested that most same-sex dyads have established and stable dominance structures. In the first study, 46% of the cages in our colony had bite wounds: 45% of same-sex males, 50% of same-sex females, and 40% of mixed-sex breeders. Females in same-sex housing had significantly less severe wounds than males, but females inflicted all the wounds in mixed-sex housing. In the second study, same-sex male and female cages chased each other 10% of the night, while no fighting was observed in aged female cages. The cages that fought had mostly asymmetric (i.e., unidirectional) and stable dominance structures. In the remainder of this section, we discuss some limiting conditions on this evidence and consider how these findings compare to similar studies on *Mus* and other laboratory-housed rodents.

Two features of these studies limit the conclusions we can draw about bite wounding, fighting, and dominance structures in laboratory-housed *Acomys*. First, the bite wounding study and the dominance studies were separate and did not include the same animals, making it implausible to determine the relationship between bite wounding and dominance structures. Second, although the home-cage video observations in the second study were robust, they suffered from a low number of cages for each housing group and excluded mixed-sex housing. Mixed-sex housing was excluded due to the presence of breeding and pups, but the bite wounding study suggests that dominance structures and fighting in mixed-sex housing are comparable to same-sex housing despite the presence of pups.

Our findings on the incidence of bite wounding in *Acomys* contrast with reports on bite wounding in laboratory-housed *Mus*. Around 1.5–14% of *Mus* cages have bite wounds, making it a major welfare concern [10,11,12]. In this study, we report that 46% of *Acomys* have bite wounds likely due to agonistic encounters and thus is a major welfare concern. In *Mus*, most bite wounds are observed in same-sex male cages; however, in *Acomys,* we found that same-sex male, female, and mixed-sex housing led to similarly high levels of bite wounding. Thus, exclusively studying one sex or group composition will likely not minimize this high incidence of bite wounding. Testing housing strategies that have been shown to alleviate bite wounding in *Mus* is warranted [15,41,42,43,44], albeit it appears difficult to fully eliminate bite wounding in *Mus* [6].

Our findings on the incidence of fighting behaviors in *Acomys* also contrast with reports on fighting in laboratory-housed *Mus*. Studies on established and stable groups of *Mus* suggest that they fight for around 1% of their nocturnal, active time [16,45], while the *Acomys* in our study engaged in fighting behaviors for 10% of the time during the same period. We specifically studied adult *Acomys* housed since weaning to increase the chance of observing stable dominance structures [46]. We also observed similar levels of fighting behaviors in same-sex males and females, which is unusual compared to *Mus*. Overt or active fighting behaviors like chasing, attacking, or biting can be instigated in female *Mus* following food deprivation [47] or applying androgen odorants to their anogenital area [13]. However, female *Acomys* appear to have similar levels of fighting behaviors compared to *Mus* without instigation. Thus, the mechanisms underlying their aggressive behaviors are likely similar between the sexes, albeit females appear to be dominants in mixed-sex housing [21]. This lack of a sex difference could be compared to Syrian hamsters (*Mesocricetus auratus*) or domesticated hedgehogs (*Atelerix albivenris*), where females are known to attack and kill males, leading to single-housing [48,49]. Albeit *Acomys* are routinely housed in groups in our colony, and killing is rare. Also, a recent study notes that virgin male and virgin female rats show similar levels of appetitive aggression when given the opportunity to engage in a same-sex resident/intruder assay [50]. Thus, sex differences in rodent fighting behavior may be specific to mice and not generalized to other rodents (e.g., *Acomys*, hamsters, hedgehogs, and rats).

In opposition to the frequent fighting and bite wounding, most of the *Acomys* in our second study had asymmetric and stable dominance structures. Typically, more frequent fighting behaviors indicate that the dominance structure is unstable, with groupmates frequently switching dominance ranks [51]. The more frequent fighting behaviors in *Acomys* may be evidence of the animals enforcing their dominance or territory, which may be unnecessary in older animals. However, the animals still huddled with each other rather than the subordinate completely avoiding the dominant. So, the exact nature of the dominant–subordinate relationship remains unclear. Future studies should consider fighting behaviors across ontogeny in mixed-sex housing and what might separate groups with frequent and infrequent fighting behaviors despite stable dominance structures.

The only housing condition that had no fighting was the aged females. In the second study, the aged females were 23 to 34 months of age and were never observed fighting in same-sex housing. The first study also included some aged animals (18 to 23 months at the start of observation), who never wounded each other. Fighting behaviors are known to decline with age in male rats and mice at around 23 months [52,53]. Whether fighting behaviors can be instigated in aged *Acomys* to determine their dominance structure remains unclear. Overall, the mechanisms underlying aggression may have an age-related decline in male *Mus* and rats and female *Acomys*.

High levels of bite wounding and fighting with stable dominance structures in *Acomys* may impact research findings if not considered in study designs, but specific research is necessary on this topic. Bite wounding itself is a major concern for experiments if it causes attrition in the experiment, and studies in *Mus* suggest that bite wounds may alter the innate immune system [7], which is central to scar-free tissue regeneration in *Acomys* [8]. Bite wounds may also alter menstrual cycling in *Acomys* since physical trauma in humans is known to lead to irregular patterns in menstrual cycling [9]. Research on dominance structures in *Mus* has also shown that the type of dominance structure and the rank position of the animal can affect behaviors and stress physiology in standard experimental assays [54,55,56]. Thus, studies on *Mus* suggest that the bite wounding and dominance structures reported in this study could impact current *Acomys* research.

## 5. Conclusions

In summary, we report that 46% of *Acomys* cages, regardless of sex, had bite wounds throughout one year of observation and that both sexes engaged in frequent low levels of fighting despite stable dominance structures. This study provides the groundwork for future studies aimed at decreasing bite wounds and fighting in *Acomys* by manipulating housing and care strategies. This high level of bite wounding is likely a major welfare concern despite the remarkable ability of *Acomys* to heal these wounds scar-free. It also has the potential to negatively impact scientific experiments, but follow-up research for these studies is necessary. Most importantly, this study clearly demonstrates that female *Acomys* wound and fight one another, contrasting with the predominant male fighting in *Mus*, thereby necessitating housing strategies and experimental considerations for both sexes.

## Figures and Tables

**Table 1:** Wound severity score sheet.

Metric	Wound Observation	Score
Character of Wound	No wound	0
	Single laceration	1
	Single circular wound	2
	Single freeform/undefined or multiple wounds	3
Surface Area	0%	0
	1%	1
	2%	2
	≥3%	3
Deepness of Wound	No lesion	0
	Superficial	1
	Deep, muscle exposed	3
Region	No lesion	0
	Anterior or Posterior (Waves or Dots)	1
	Anterior and Posterior (Waves and Dots)	2
	Face or Genitals (Checkers)	3
Total		12

A score of 10 or higher meets the criteria for a humane endpoint.

**Table 2:** Behavioral ethogram.

Category	Behavior	Description
Offensive Agonistic Behavior	Chase *	Actor mouse rapidly pursues target mouse while target mouse flees, unaccompanied by mounting
	Mount	Actor mouse rapidly pursues target mouse while target mouse flees, accompanied by side or rear mounting
	Attack	Actor mouse lunges and/or bites target mouse without target mouse counterattacking
	Food steal	Target mouse has control of food by holding it with both forepaws and/or mouth. Actor mouse then takes control of food, leaving target mouse without control
Defensive Agonistic Behavior	Flee	The actor mouse approaches the target mouse, and the target mouse rapidly flees; the actor follows by chasing or mounting
	Displace *	The actor mouse approaches the target mouse, and the target rapidly flees; the actor does not follow
	Freeze	Target mouse is immobile in response to agonistic offensive behavior of actor mouse
Huddling **	Side-huddle **	One mouse is resting in direct body contact side-by-side
	Mounted-huddle **	One mouse is resting with at least their forepaws on the dorsal side of the other mouse
Other	Active **	Animal is observable and performing any activity for at least 5 s; must see entire head of mouse
	Inactive	Mouse visible and motionless for >15 s
	Unseen	Cannot reliably code whether mouse is in or on a shelter

* denotes the behavior recorded for both one-zero and focal animal sampling while ** denotes the behavior was restricted to one-zero sampling and was not measured during focal animal sampling.

**Table 3:** Frequency of wins and dominance structure.

Cage	Condition	Wins by Dominant	Wins by Subordinate	Directional Consistency	Structure
A	Male Pairs	4	0	1	Asymmetric
B		159	0	1	Asymmetric
C		243	0	1	Asymmetric
D	Female Pairs	58	35	0.25	Symmetric
E		3	0	1	Asymmetric
F		276	0	1	Asymmetric
G	Aged Females	0	0	0	Unmeasurable
H		0	0	0	Unmeasurable
I		0	0	0	Unmeasurable

## Data Availability

All codes and additional materials can be found in the following GitHub repository: https://github.com/javarhol/Acomys_Bitewound_Dominance_2023.

## Supplementary Materials

The following supporting information can be downloaded at https://www.mdpi.com/article/10.3390/ani14010064/s1. Figure S1: Activity heat-map across three weeks. A subset of young *Acomys* was used to measure the frequency of activity across 72 h every week for 3 weeks. Activity was measured using one-zero coding every minute for the first 15 min of each hour; Table S1: Experimental timeline; Table S2: Kruskal–Wallis results for wound-severity metrics; Figure S2: Age has no relation to bite wounding. A: Bar plot comparing wounded and not wounded *Acomys* in relation to age in months. Bars represent the mean age for the wounded and not wounded; error bars are the standard error of the mean. Points represent individual animals. B: Line and scatter plot comparing total wound score and age in months. Points represent individual animals, and the line is an estimated linear model from the point values; Table S3: Kruskal–Wallis results of behaviors measured during one-zero coding; Table S4: Frequency of wins each week for each cage.
