# Supplementary material for: Bite Wounds and Dominance Structures in Male and Female African Spiny Mice (Acomys cahirinus): Implications for Animal Welfare and the Generalizability of Experimental Results
Source: Animals (Basel). 2023 Dec 23;14(1):64. doi: 10.3390/ani14010064 (PMC10778049; doi:10.3390/ani14010064)
Supplement: Supplementary file 1 [file animals-14-00064-s001.zip › Table S1.pdf]

Table S1: Experimental Timeline

Day 1	Day 2	Day 3	Day 4	Day 5	Day 6	Day 7
Cage-Change	1/0 sampling	1/0 sampling	1/0 sampling			
Special Diet	Focal sampling	Focal sampling	Focal sampling			
**Day 8**	**Day 9**	**Day 10**	**Day 11**	**Day 12**	**Day 13**	**Day 14**
	1/0 sampling	1/0 sampling	1/0 sampling			
Special Diet	Focal sampling	Focal sampling	Focal sampling			
**Day 15**	**Day 16**	**Day 17**	**Day 18**	**Day 19**	**Day 20**	**Day 21**
Cage-Change	1/0 sampling	1/0 sampling	1/0 sampling			
Special Diet	Focal sampling	Focal sampling	Focal sampling			

*1/0 sampling and focal sampling started at 20:00h, the start of the dark-cycle
