# Supplementary material for: Bite Wounds and Dominance Structures in Male and Female African Spiny Mice (Acomys cahirinus): Implications for Animal Welfare and the Generalizability of Experimental Results
Source: Animals (Basel). 2023 Dec 23;14(1):64. doi: 10.3390/ani14010064 (PMC10778049; doi:10.3390/ani14010064)
Supplement: Supplementary file 1 [file animals-14-00064-s001.zip › Table S2.pdf]

Table S2: Kruskall-Wallis results for wound severity metrics

Wound Metric	Chi-squared	df	p-value
**Surface Area**	1.713	2	0.425
**Character**	4.283	2	0.118
**Deepness**	0.987	2	0.611
**Region**	9.525	3	0.009
