# Supplementary material for: Bite Wounds and Dominance Structures in Male and Female African Spiny Mice (Acomys cahirinus): Implications for Animal Welfare and the Generalizability of Experimental Results
Source: Animals (Basel). 2023 Dec 23;14(1):64. doi: 10.3390/ani14010064 (PMC10778049; doi:10.3390/ani14010064)
Supplement: Supplementary file 1 [file animals-14-00064-s001.zip › Table S3.pdf]

Table S3: Kruskal-Wallis results of behaviors measured during one-zero coding

**Behavior**	**Chi-squared**	**df**	**P-value**
**Activity**	5.956	2	0.051
**Chasing**	5.793	2	0.055
**Displace**	5.793	2	0.055
**Mounting (active)**	5.844	2	0.054
**Side Huddle**	6.489	2	0.039
**Mounted Huddle**	5.600	2	0.061
