# Supplementary material for: Bite Wounds and Dominance Structures in Male and Female African Spiny Mice (Acomys cahirinus): Implications for Animal Welfare and the Generalizability of Experimental Results
Source: Animals (Basel). 2023 Dec 23;14(1):64. doi: 10.3390/ani14010064 (PMC10778049; doi:10.3390/ani14010064)
Supplement: Supplementary file 1 [file animals-14-00064-s001.zip › Table S4.pdf]

Table S4: Frequency of wins each week for each cage

Housing condition	Cage	Week1	Week2	Week3	Stability Category
Young Male Pairs	A	4	0	0	Static+Infrequent
	B	86	42	31	Static+Frequent
	C	138	90	15	Static+Frequent
Young Female Pairs	D	37	27	29	Dynamic+Frequent
	E	3	0	0	Static+Infrequent
	F	95	138	43	Static+Frequent
Aged Female Pairs	G	0	0	0	Unmeasurable
	H	0	0	0	Unmeasurable
	I	0	0	0	Unmeasurable
