# Supplementary figures and images for: Bite Wounds and Dominance Structures in Male and Female African Spiny Mice (Acomys cahirinus): Implications for Animal Welfare and the Generalizability of Experimental Results
Source: Animals (Basel). 2023 Dec 23;14(1):64. doi: 10.3390/ani14010064 (PMC10778049; doi:10.3390/ani14010064)

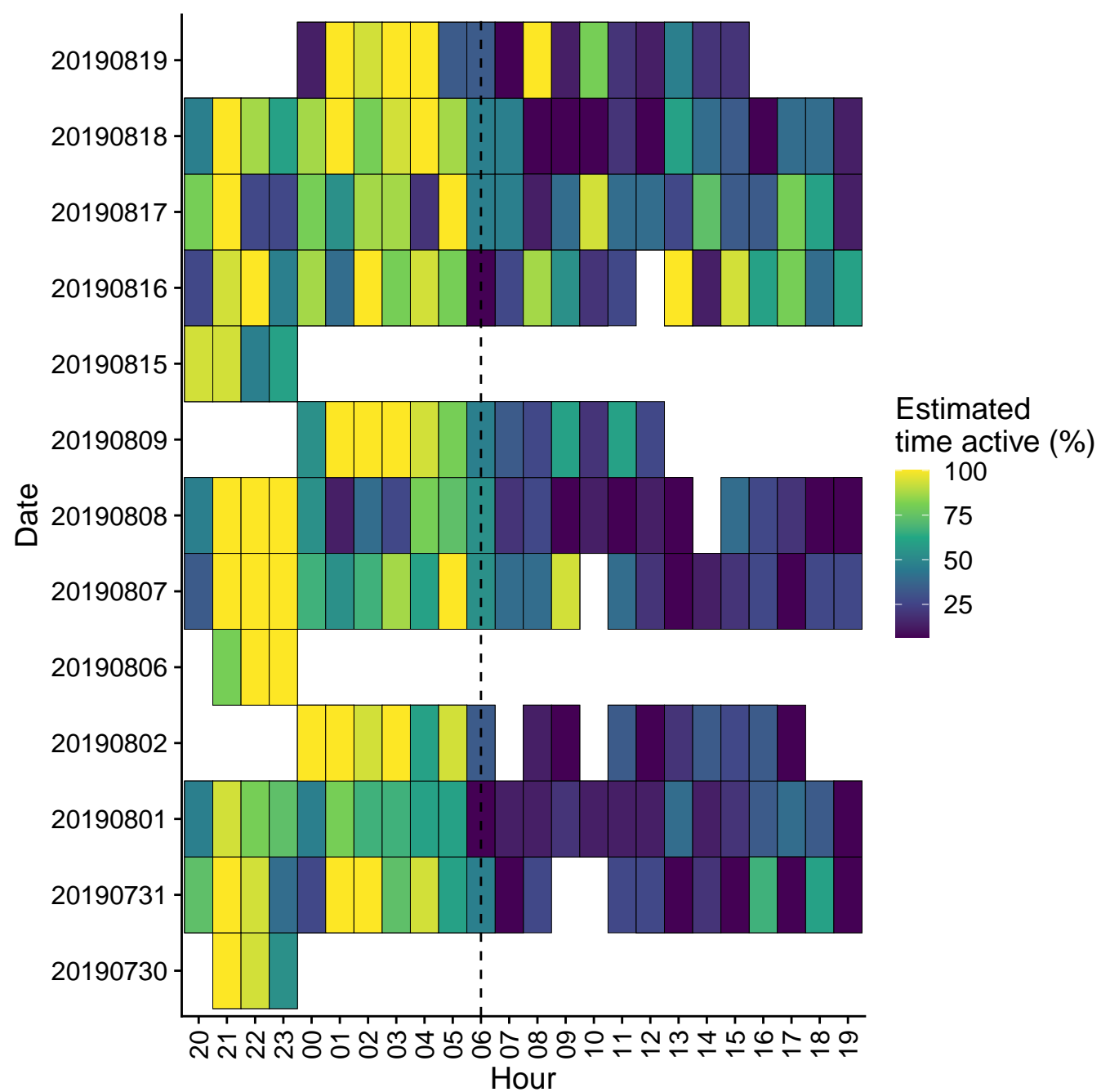

Supplement: Supplementary file 1 [file animals-14-00064-s001.zip › Figure S1.pdf]

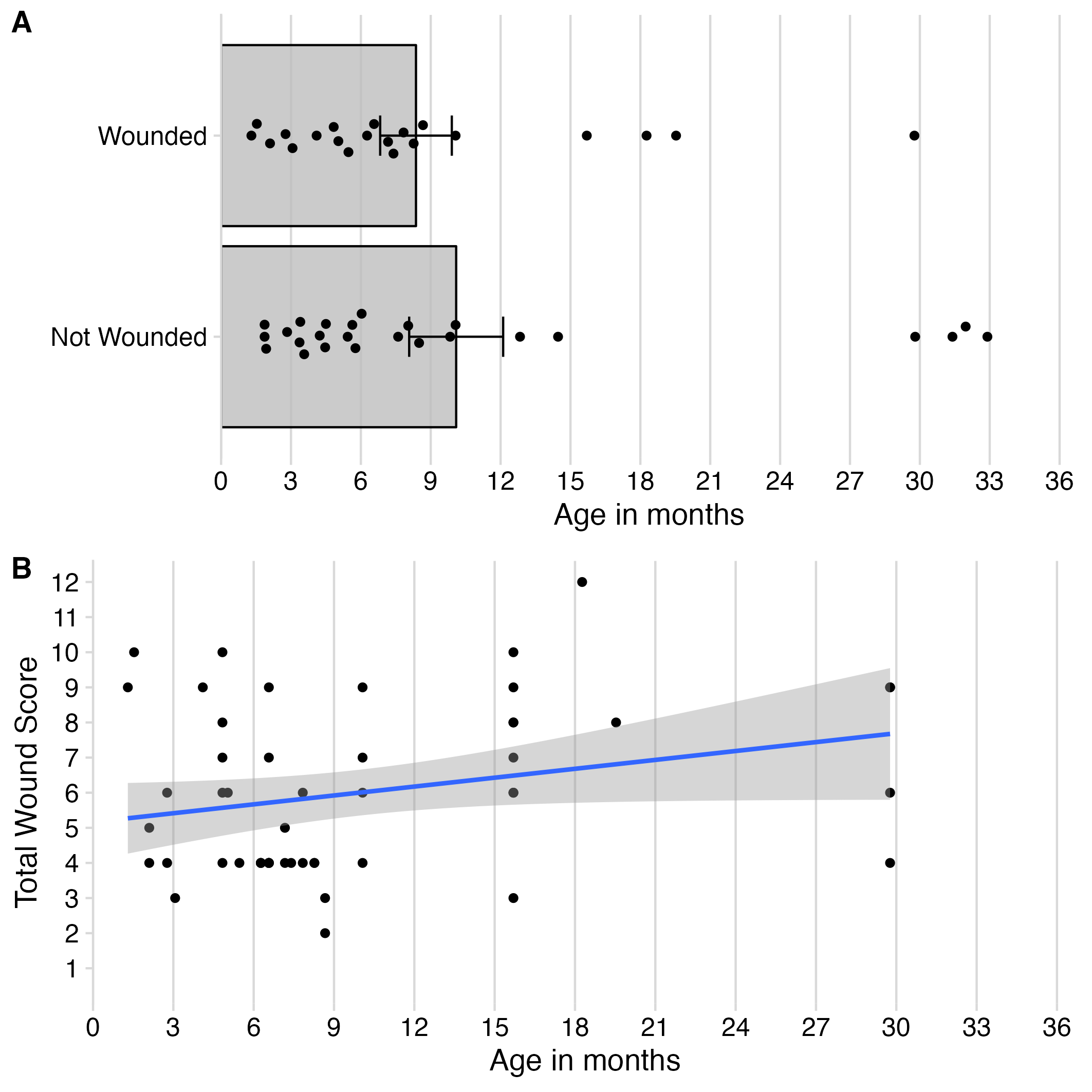

Supplement: Supplementary file 1 [file animals-14-00064-s001.zip › Figure S2_AgeWound.png]
